# Environmental Occurrence, Influencing Factors, and Toxic Effects of 6PPD-Q

**DOI:** 10.3390/toxics13110906

**Published:** 2025-10-22

**Authors:** Tengwen Yin, Ying Liang, Yanju Liu, Jia Liu, Xuedong Wang

**Affiliations:** 1 College of Resource Environment and Tourism, Capital Normal University, Beijing 100048, China; 2240902071@cnu.edu.cn; 2 Institute of Analysis and Testing, Beijing Academy of Science and Technology (Beijing Center for Physical & Chemical Analysis), Beijing 100089, China; liangying@bcpca.ac.cn (Y.L.); liuyanju@bcpca.ac.cn (Y.L.)

**Keywords:** 6PPD-Q, emerging contaminants, environmental fate, influencing factors, bioaccumulation, human health

## Abstract

The antioxidant *N*-(1,3-dimethylbutyl)-*N*′-phenyl-*p*-phenylenediamine (6PPD) is widely incorporated into tires to extend their service life. However, in the presence of ozone, it is readily transformed into *N*-(1,3-dimethylbutyl)-*N*′-phenyl-*p*-benzoquinone (6PPD-Q). Owing to the large-scale production and widespread utilization of rubber-related products, 6PPD-Q is continuously released into the environment with tire and road wear particles, becoming ubiquitous across multiple environmental compartments. It possesses bioaccumulation potential and exhibits significant toxicity, while multiple exposure pathways enable it to enter human body, posing risks to public health. This review summarizes the environmental distribution of 6PPD-Q in atmospheric, aquatic, and terrestrial systems, and examines key factors influencing its occurrence, including precipitation patterns, traffic characteristics, sunlight, and particle size. The toxicological effects of 6PPD-Q are also discussed. Based on these findings, a comprehensive management framework encompassing “source reduction—process regulation—end-of-pipe treatment” is proposed. Finally, current knowledge gaps are identified and future research directions are highlighted.

## 1. Introduction

As urbanization continues to accelerate globally, emerging contaminants have emerged a significant threat to both ecosystems and public health. Every year, approximately 5.9 million tons of tire wear particles (TWPs) are released into the environment, with 20% eventually reaching aquatic ecosystems [1]. TWPs constitute a major source of non-exhaust emissions, and their quantity is steadily increasing alongside the growing global vehicle population [2]. The antioxidant *N*-(1,3-dimethylbutyl)-*N*′-phenyl-*p*-phenylenediamine (6PPD) is commonly incorporated into rubber tires to prevent oxidation [3]. However, 6PPD can be easily oxidized to form *N*-(1,3-dimethylbutyl)-*N*′-phenyl-*p*-benzoquinone (6PPD-Q). 6PPD and 6PPD-Q may also originate from various rubber-based goods, including sports gear, recreational products, and rubber tubing [4]. Due to their widespread use, both 6PPD and 6PPD-Q have been identified as pervasive contaminants in various environmental media [5], including the atmosphere [6], water [7], soil [8], and sediments [9].

Since the landmark 2021 study in *Science* [10] identified 6PPD-Q as the second most toxic emerging contaminant to aquatic life, its occurrence and toxicity have raised considerable global concern [11,12]. Urban stormwater runoff is recognized as the major pathway through which 6PPD-Q enters and degrades surface water ecosystems [13], a process worsened by increasing extreme rainfall events associated with global climate change. The widespread distribution of 6PPD-Q enables its uptake by plants and animals, ultimately threatening human health at the food chain [12]. These findings have drawn growing international attention to 6PPD-Q and positioned it as an emerging issue in environmental policy discussions rather than an established global priority. For example, the United States has listed its precursor, 6PPD, as a hazardous substance under the Toxic Substances Control Act [14], and Washington State passed the Safer Products Act (Senate Bill 5931) in 2024, mandating that all motor vehicle tires containing 6PPD undergo comprehensive review and control by the State’s Department of Ecology [15]. California’s Safer Consumer Products Regulations also require tire manufacturers to conduct alternatives analyses for 6PPD in tires sold statewide [16]. In China, although the tire industry has made strides toward emission reductions through policies like the Evaluation Requirements for Tire Industry Green Factories [17], specific regulations targeting 6PPD or 6PPD-Q are still lacking.

This review conducted a comprehensive literature search across major scientific databases, including Web of Science, Scopus, and PubMed, using keywords such as *N*-(1,3-dimethylbutyl)-*N*′-phenyl-*p*-phenylenediamine (6PPD), *N*-(1,3-dimethylbutyl)-*N*′-phenyl-*p*-phenylenediamine quinone (6PPD-Q, also referred to as 6PPD-quinone), *p*-phenylenediamines (PPDs), PPD-quinones (PPD-Qs), tire wear particles (TWPs), occurrence, environmental risks, bioaccumulation, biotransformation, ecotoxicity, aquatic and terrestrial organisms, human health, and runoff. The search period was restricted to studies published between 2021 and 2025 to capture the most recent research following the landmark identification of 6PPD-Q’s toxicity in *Science* [10]. After removing duplicates, the literature screening was performed based on titles and abstracts to exclude irrelevant studies, publications with unclear experimental design, flawed data analysis, non-peer-reviewed sources, or insufficient methodological details, thereby ensuring scientific rigor. Eligible studies then underwent a full-text assessment based on predefined inclusion criteria. Additionally, the reference lists of key articles were examined to identify additional sources. The core content and findings from the included studies were systematically extracted and synthesized for this review.

The aim of this review was threefold: (1) to synthesize the current knowledge on the environmental distribution, transformation, and toxic effects of 6PPD-Q across multiple systems; (2) to identify key environmental drivers influencing its occurrence; and (3) to propose a comprehensive management framework. Despite the rapid growth in research since 2021, significant gaps remain regarding its long-term fate of 6PPD-Q, its bioaccumulation potential, human exposure risks, and effective mitigation strategies. This review seeks to integrate current evidence, clarify driving mechanisms, and support the development of holistic risk management strategies for this emerging contaminant.

## 2. Environmental Fate of 6PPD-Q

TWPs are ubiquitous environmental constituents and emerging contaminants, with per-person emissions estimated to range from 0.23 to 1.9 kg/year globally [18]. 6PPD is primarily added to tires to prevent wear and maintain long-term structural integrity [19]. However, it has significant potential to react with ozone, forming its derivative 6PPD-Q, which is a toxic compound [20]. As newly identified environmental pollutants, both 6PPD and 6PPD-Q have been consistently documented across multiple environmental compartments, including water [5,13,19,21,22,23], atmosphere [3,23,24], dust [4,24,25], and soil [23].

**Figure 1:**
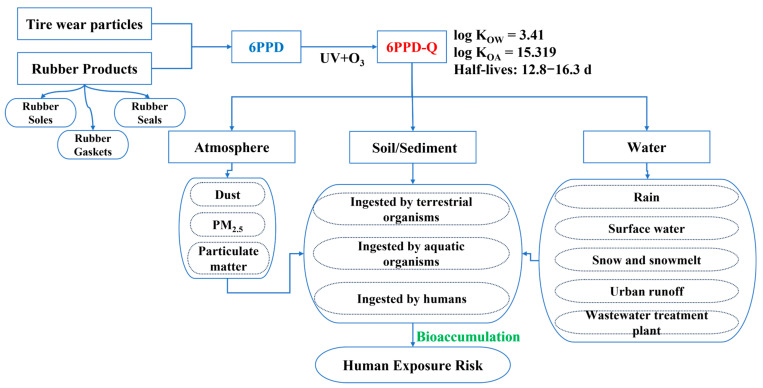
Environmental fate, transformation, and risk of 6PPD-Q.

The mobility and persistence of 6PPD-Q vary across different environmental compartments and are strongly influenced by its physicochemical properties. Compared with its parent compound 6PPD, which undergoes hydrolysis in aqueous environments with half-lives ranging from 4.8 to 64 h, 6PPD-Q is more persistent, exhibiting half-lives of 12.8–16.3 d in water [26]. Its low vapor pressure (2.03 × 10^−4^ Pa at 25 °C) and high octanol-air partition coefficient (log K_OA_) (15.319 > 10) indicate minimal volatility and a strong tendency to adsorb onto atmospheric particles, thereby limiting long-range atmospheric transport while enhancing deposition into other environmental compartments [27]. Furthermore, its octanol–water partition coefficient (log K_ow_ = 3.41, data from ChemSpider) suggests a considerable potential for bioaccumulation in living organisms under natural conditions, see Figure 1.

### 2.1. Atmosphere

PPDs and PPD-Qs exhibit widespread distribution across different types of atmospheric particulate matter, including PM_2.5_ and dust. Their detection frequencies range from non-detectable to 100%, while concentrations spanning from pg/m^3^ to ng/m^3^. For example, monitoring data from Guangzhou, China, documented 6PPD-Q concentrations ranging from 2.96 to 7250 pg/m^3^ in PM_2.5_ [3] and 4.02–2369 ng/g in dust samples [28]. Table 1 summarizes the atmospheric concentrations of 6PPD-Q.

**Table 1:** Atmospheric concentrations of 6PPD-Q.

Type of Sample	Sampling Sites	Sample Size	Concentration	Reference
Dust	Tokyo, Japan	22	116 ng/g	[1]
	Guangzhou, China	59	<LOD–277 ng/g	[4]
	Guangzhou, China	60	4.02–2369 ng/g	[28]
	Beijing, China	30	--	[29]
	Shanghai, China	--	19.1 ng/g	[30]
	Nanjing, China	--	168.1 ng/g	[30]
Dust	Hangzhou, China	--	201.4 ng/g	[30]
	Görlitz, Germany	2	220–270 ng/g	[31]
	Guangzhou, China	60	10.5–109 ng/g	[28]
	Hong Kong, China	12	9.50–936 ng/g	[23]
	Sao Paulo, Brazil	--	1.75 pg/m^3^	[32]
	Buenos Aires, Argentina	--	1.27 pg/m^3^	[32]
	Bogota, Colombia	--	0.68 pg/m^3^	[32]
	Sydney, Australia	--	0.17 pg/m^3^	[32]
Dust from electronic waste	South China	45	87.1–2850 ng/g	[33]
PM_2.5_	Guangzhou, China	48	2.96–7250 pg/m^3^	[3]
	Taiyuan, China	24	2.44–1780 pg/m^3^	[3]
	Taiyuan, China	24	1.1–84 pg/m^3^	[34]
	Zhengzhou, China	12	0.3–32 pg/m^3^	[34]
	Shanghai, China	8	0.3–39 pg/m^3^	[34]
	Nanjing, China	6	1.1–68 pg/m^3^	[34]
	Hangzhou, China	7	0.8–26 pg/m^3^	[34]
	Guangzhou, China	24	0.1–15 pg/m^3^	[34]
Particulate matter in the atmosphere	Guangzhou, China	124	0.16–39.2 pg/m^3^	[24]
	Hong Kong, China	18	0.54–13.8 pg/m^3^	[23]

Note: LOD: limit of detection; --, not provided in the reference.

Using a global passive air sampling network, Johannessen et al. [32] identified 6PPD-Q in dust across 18 megacities worldwide, with concentrations ranging from below detection limits to 1.75 pg/m^3^. Among these, São Paulo had the maximum level (1.75 pg/m^3^), while progressively lower concentrations were recorded in Buenos Aires (1.27 pg/m^3^), Bogota (0.68 pg/m^3^), and Sydney (0.17 pg/m^3^) [32]. In China, 6PPD-Q has also been detected in multiple regions. For instance, measurements from four waste recycling facilities in Guangzhou reported concentrations of 0.16–39.2 pg/m^3^ [24]. Furthermore, A nationwide survey by Zhang et al. [30] confirmed its ubiquitous presence in urban road dust, with a 100% detection frequency across 55 major Chinese cities. Regional analysis showed the highest concentrations in Changchun (349 ng/g), with Lanzhou (266 ng/g) and Hangzhou (201 ng/g) ranking next, highlighting the role of industrial-scale rubber tire production and wear in driving PPD release to urban environments. Notably, Changchun—home to China’s first automobile manufacturer and a long-standing automotive production hub—showed the highest levels, reflecting its strong linkage between automotive industry activity and environmental PPD contamination [35].

In addition to dust, 6PPD-Q has been consistently identified as a component of PM_2.5_ pollution. Zhang et al. [34] were the first to report multiple PPDs in PM_2.5_ across six major Chinese cities (Taiyuan, Zhengzhou, Shanghai, Nanjing, Hangzhou, and Guangzhou). In 2019, vehicle populations in these cities were 155.3 × 10^4^, 381.3 × 10^4^, 415.8 × 10^4^, 270.2 × 10^4^, 267.7 × 10^4^, and 280.3 × 10^4^, respectively. Detected compounds included 6PPD, *N*-isopropyl-*N*′-phenyl-*p*-phenylenediamine (IPPD), *N*,*N*′-di-sec-butyl-*p*-phenylenediamine (44PD), *N*-(1,4-dimethylpentyl)-*N*′-phenyl-*p*-phenylenediamine (77PD), *N*-cyclohexyl-*N*′-phenyl-*p*-phenylenediamine (CPPD), *N*,*N*′-diphenyl-*p*-phenylenediamine (DPPD), and *N*,*N*′-di(2-naphthyl)-*p*-phenylenediamine (DNPD). Most PPDs were detectable in all samples, with the median ∑PPDs concentration in Guangzhou (103 pg/m^3^) approximately twice those measured in Taiyuan (59 pg/m^3^), Nanjing (51 pg/m^3^), and Zhengzhou (46 pg/m^3^) [34]. A moderate correlation (*R* = 0.4) was observed between the annual median concentrations of PM_2.5_-bound PPDs and city-specific vehicle numbers, suggesting that vehicular emissions play a significant role in determining PPD pollution levels [34]. Taiyuan, however, deviated from this trend, likely due to distinct regional characteristics and industrial structures [3]. Similarly, Wang et al. [3] reported higher median ∑PPDs (3220 pg/m^3^) and 6PPD-Q (1100 pg/m^3^) concentrations in Guangzhou compared with Taiyuan (1150 pg/m^3^ and 744 pg/m^3^, respectively), further supporting the influence of vehicle density on atmospheric PPD contamination. Moreover, 6PPD-Q levels in PM_2.5_ were found to increase proportionally with O_3_ and precursor 6PPD concentrations [3,34], consistent with the ozonation-driven conversion pathway proposed by Cao et al. [23], in which 6PPD reacts with atmospheric O_3_ to generate toxic 6PPD-Q.

### 2.2. Water

When tires interact with road surfaces through friction, 6PPD-Q can be deposited on roads and subsequently transported into near water bodies via surface runoff [36], with typical concentrations ranging from μg/L to mg/L. Rainwater samples show pronounced spatial variability in 6PPD-Q levels, with higher concentration observed in China (0.21–2.43 μg/L) [11] and the United States (0.002–0.29 μg/L) [37] compared to Canada (0.086–1.4 μg/L) [13]. Moreover, both 6PPD and 6PPD-Q are ubiquitous in various environmental media, including snow and snowmelt runoff [10,13,19], wastewater treatment plants (WWTPs) [19], and river water [21,22,38]. A summary of 6PPD-Q concentrations in different aquatic environments is presented in Table 2.

**Table 2:** Concentrations of 6PPD-Q in different water bodies (ng/L).

Type of Sample	Sampling Sites	Sample Size	Concentration	Reference
Urban runoff	Hong Kong, China	9	9.50–936	[23]
	Jiaojiang, China	--	<LOD–21	[39]
	Seattle, USA	16	800–19,000	[10]
	USA	4	67–233	[40]
Rain	Saskatoon, Canada	21	86–1400	[13]
Surface water	Don River, Canada	29	300–2300	[22]
	Don River, Canada	21	110–540	[38]
	Highland Creek, Canada	21	210–720	[38]
	Brisbane River, Australia	32	0.38–88	[21]
	Miller Creek, USA	5	46–110	[40]
Snow and snowmelt	Saskatoon, Canada	32	15–756	[13]
WWTPs	Leipzig, Germany	22	0–105	[19]

Note: LOD: limit of detection; --: not provided in the reference.

Under dry weather conditions, the total load of 6PPD-Q was estimated at 5 g/d, which increased sharply to 17 g/d during snowmelt and 26 g/d during rainfall events [19]. The detection frequency of 6PPD-Q in runoff samples reached up to 100%. The major sources of 6PPD-Q in urban runoff include: (1) leaching from TWPs that was washed into rivers through precipitation and road sweepings [41,42]; (2) deposition (both dry and wet) of atmospheric particulate matter containing 6PPD-Q [1,37]; (3) photochemical transformation of 6PPD in sunlit aquatic environments [43,44].

Road runoff analysis from the U.S. West Coast revealed 6PPD-Q concentrations ranging from 0.8 to 9 μg/L [10]. Levels measured in Seattle (800–19,000 ng/L) and Los Angeles (4100–6100 ng/L) were markedly higher than those in Hong Kong’s urban runoff (210–2430 ng/L) [10,23]. In a study comparing multiple environmental matrices (rainwater, snowmelt, and solid snow), Challis et al. [13] reported higher mean concentrations in rainwater (593 ng/L) than in snowmelt (367 ng/L), suggesting that both snowmelt and rainfall contribute to elevated levels of 6PPD-Q. Similarly, Seiweirt et al. [19] found that more than 90% of 6PPD-Q in urban road snow was predominantly associated with the particulate phase rather than the aqueous phase. During snowmelt and rainfall events, 6PPD-Q enters the river through urban road runoff, posing increased risks to aquatic ecosystems [19,22]. Increased surface runoff due to urbanization further enhances the transport of tire-wear-related contaminants. Global surveys have confirmed the widespread occurrence of 6PPD-Q in rivers, with concentrations ranging from 0.26 to 11.3 ng/L in China, 15–756 ng/L in Canada, 110–428 ng/L in German [45,46]. 6PPD-Q is also frequently detected in wastewater treatment plants (WWTPs). In Guangzhou, China, influent concentrations ranged from 14.2 to 69.8 ng/L [47], while a mean concentration of 105 ng/L was reported for influent samples from a German WWTP [19]. These levels are generally lower than those found in surface runoff, likely due to dilution by domestic wastewater. Interestingly, detection frequencies in WWTP effluents from China were higher than in the influents, possibly due to the release of 6PPD-Q from TWPs, conversion of 6PPD during treatment, or the persistence of 6PPD-Q throughout the treatment process [48].

Given its persistence and mobility, there is a pressing need to improve existing wastewater treatment technologies or develop advanced strategies to efficiently remove 6PPD-Q from aquatic systems. Promising strategies include advanced oxidation (e.g., UV-activated peroxymonosulfate) [49], disinfection (hypochlorite and chlorine dioxide) [50], microbial degradation [51], and adsorption using engineered nanomaterials [52].

While substantial research has characterized the distribution of 6PPD-Q in urban runoff, most studies have focused primarily on its aqueous fraction, overlooking its particulate-bound form. As indicated previously, dry deposition is a major pathway through which 6PPD-Q enters aquatic systems via contaminated particulate matter (e.g., dust and PM_2.5_). Consequently, studies of 6PPD-Q considering only the dissolved phase may underestimate total 6PPD-Q pollution. Therefore, to more accurately understand the occurrence characteristics of 6PPD-Q in water bodies, future studies should focus on its partitioning between dissolved and particulate phases in rainfall runoff, as well as its long-term effects on aquatic ecosystems, such as chronic toxicity and bioaccumulation in aquatic food webs, impacts on sediment-dwelling organisms, and potential disruptions to key ecosystem functions. As major sinks and potential secondary sources of 6PPD-Q, sediments will be discussed in detail alongside soils in the following section.

### 2.3. Sediment/Soil

Sediments act as major reservoirs for the transport, transformation, and long-term storage of pollutants, accumulating diverse organic contaminants. Analyzing sediment-associated 6PPD-Q is crucial for tracing pollution sources and exposure pathways. Comprehensive surveys have confirmed its pervasive presence in both marine and freshwater environments, with hotspots in China including the Pearl River Delta, Yangtze River Delta, South China Sea coastal waters, and even deep-sea regions [53]. Total concentrations of PPDs and PPD-Qs in sediments displayed a distinct spatial decreasing pattern, with median values of 39.7 and 15.2 ng/g in urban rivers, declining sequentially to 14.0 and 5.85 ng/g in estuaries, 9.47 and 2.97 ng/g in coastal areas, and the lowest levels (5.24 and 3.96 ng/g) in deep-sea sediments. Concurrently, a gradual decline in the 6PPD/6PPD-Q ratio was observed along the land-to-sea transect, decreasing from 1.5 to 1.0, which reflects the greater stability of 6PPD-Q and its continuous formation during long-distance transport. Hydrodynamic conditions of coastal and ocean currents further influence its distribution and deposition.

Region-specific inputs further elevate sediment burdens. For example, 6PPD-Q levels in the Jiaojiang River (median 2.8 ng/g) were comparable to the upper range in the Pearl River Delta (0.82–9.03 ng/g) and significantly exceeded those in the Pearl River Estuary (<LOD–2.0 ng/g) [39]. Besides inputs from urban stormwater and road runoff, local sources such as rubber tires hung on ships and dock edges, together with heavy shipping traffic, are likely to contribute to elevated PPD levels in this river [39]. Similarly, in South Korea, transformation of 6PPD from urban runoff and road dust has led to 6PPD-Q concentrations up to 38 ng/g in sediments of inland streams and Lake Sihwa, an area strongly influenced by industrial and traffic activities [54]. Acting as long-term sinks, sediments may also become secondary sources through pollutant remobilization, thereby sustaining ecological risks.

Soils represent another major sink for TWPs and their associated contaminants, including 6PPD, IPPD, CPPD, and their quinone derivatives. High levels have been observed in roadside soils of Hong Kong’s New Territories and Kowloon, with median concentrations of 309 ng/g for 6PPD and 234 ng/g for 6PPD-Q, accounting for 80% of total PPDs (381 ng/g) and 60% of total PPD-Qs (395 ng/g), respectively [23]. By comparison, analysis of soils from a legacy e-waste recycling zone in Guiyu, Shantou, revealed much lower concentrations of 6PPD-Q (0.002–4.4 ng/g) [55]. The main input pathways of soil contamination include atmospheric deposition, surface runoff, and anthropogenic activities. 6PPD-Q introduced via atmospheric deposition, surface runoff, and anthropogenic activities. For example, atmospheric deposition of 6PPD-Q can enhance mixed pollution loads, while rainy-season conditions increase its migration into soils [56]. Urban stormwater runoff further transports TWPs from roads surfaces, where sorption to organic-rich soil particles enhances persistence and reduces mobility [57]. Additionally, biosolids generated from WWTPs and reused as soil amendments represent another additional source, with multiple PPDs and PPD-Qs widely detected in Hong Kong biosolids [58].

The transformation of 6PPD and 6PPD-Q in soils is further shaped by soil redox conditions. 6PPD degrades rapidly under aerobic conditions with a half-life of only 0.7–1.5 d, but persists much longer in anaerobic soils (≈51.4 d), suggesting that submerged soils and sediments may serve as important secondary sources. In contrast, 6PPD-Q is more stable, with microbially mediated half-lives of 13.5–14.2 d under aerobic conditions [59].

Collectively, the dual role of sediments and soils as sinks and potential secondary sources highlights the necessity for systematic, risk-based monitoring and management of tire-derived contaminants in both terrestrial and aquatic environments.

## 3. Factors Influencing 6PPD-Q

### 3.1. Precipitation Patterns

Precipitation is a primary contributor to 6PPD-Q pollution in aquatic ecosystems. The characteristics of precipitation events (e.g., rainfall amount, intensity, and duration) strongly influence the observed levels of 6PPD-Q in aquatic environments, which typically range from ng/L to μg/L. Helm et al. [60] detected elevated 6PPD-Q concentrations in Toronto waterways as well as Great Lakes receptor waters during the rainy season, with concentrations ranging from 0.21 to 0.76 μg/L. Similarly, 6PPD-Q was detected in 100% of surface runoff specimens collected during rainfall from urban traffic areas in Hong Kong, China, with concentrations between 0.21 and 2.43 μg/L [23]. In Australian rivers, precipitation events led to substantial increase in 6PPD-Q concentrations—up to 21-fold higher than baseline levels [21]. The higher 6PPD-Q concentration observed in the Jiaojiang River, China, compared with the Pearl River (average 2.3 ng/L) and Dongjiang River (1.7 ng/L), China [47], has been attributed to greater inputs from urban stormwater and road runoff [39].

Further, evidence from Seiwert et al. [19] confirmed the pivotal role of precipitation: during dry weather, no 6PPD-Q was detected in any WWTP process stream, whereas during rainfall and snowmelt events, average influent concentrations increased to 105 ng/L and 52 ng/L, respectively. A positive correlation has also been reported between 6PPD-Q concentrations in surface water and total storm precipitation, indicating that higher rainfall volumes lead to elevated riverine levels of 6PPD-Q [38]. Johannessen et al. [22] observed 6PPD-Q concentrations in stream samples from the Toronto watershed that varied across pre-rainfall, rainfall, and post-rainfall phases, with concentrations rising over the duration of the event and peaking at 2.3 μg/L within 17–20 h after rainfall. Likewise, Liu et al. [61] demonstrated that low-intensity rainfall events triggered elevated PPD-Qs levels in stormwater. In the Thornton Creek watershed, 6PPD-Q was detected in all composite and discrete samples collected during a storm event, with concentrations increasing as rainfall duration extended [37]. Furthermore, snowmelt samples exhibited significantly elevated 6PPD-Q concentrations (67.4–129 ng/L)—from 3 to 6.5 times higher than those in river water samples—likely due to the deposition and persistence of TWPs in snow, followed by their prolonged release of 6PPD-Q [13,19].

### 3.2. Traffic Characteristics

Pollution from the automotive industry has intensified worldwide in recent years, largely driven by the substantial increase in vehicle usage [62]. Global tire production reached approximately 16.86 million tons in 2019, accompanied by an estimated compound annual growth rate of 5–7% [63]. The growth in global tire manufacturing and use has contributed to a steady release of TWPs into various environmental compartments via mechanical abrasion. Current estimates indicate that global TWPs emissions reach as high as 5.92 million tons annually [64,65], with the European Union and the United States contributing nearly 1.32 and 1.12 million tons per year, respectively [20,31]. The emission of 6PPD-Q derived from TWPs is also substantial. In the United States, annual 6PPD-Q emissions from TWPs have been estimated at around 1900 tons [10,41]. Research shows that a single four-wheeled vehicle can emit 140–700 g of 6PPD and 1.4–500 g of 6PPD-Q over its lifetime [22]. Consequently, with growing vehicle, environmental concentrations of 6PPD-Q are showing an increasing trend [3,34]. For instance, PM_2.5_ samples collected from Chinese cities such as Shanghai, Nanjing, Hangzhou, Guangzhou, and Zhengzhou showed that median PPD concentrations increased with local car ownership [34].

Traffic volume is another factor affecting the 6PPD-Q concentrations. Hiki et al. [1] reported that 6PPD-Q levels in road dust from Japan ranged between 116 and 1238 ng/g, significantly higher than those found in residential road dust (366–885 ng/g). Similarly, Zhang et al. [30] analyzed mixed dust samples from main roads in 55 Chinese cities and found that the highest median concentration of 6PPD-Q occurred in tunnel dust (171.9 ng/g), followed by urban arterial roads (60.6 ng/g), while suburban roads with lower traffic intensity exhibited the lowest levels (9.6 ng/g). Elevated concentrations were also detected in highway service areas (107.8 ng/g). The influence of vehicle activity was further demonstrated by comparing traffic-related sites with indoor environments. Significantly higher concentrations of 6PPD-Q were quantified in vehicle dust (17.9–146 ng/g) compared with indoor dust (<LOD–0.4 ng/g) [4,24]. Similarly, 6PPD-Q concentrations in parking lots (5.7–277 ng/g) greatly exceeded those found in indoor rooms (<LOD–0.4 ng/g) [4].

In addition to traffic density, other driving-related factors such as vehicle speed, braking, and acceleration significantly affect TWP emissions. Track tests conducted by Beji et al. [66] showed that TWPs emissions increases significantly with higher vehicle speeds, braking force, and acceleration, consistent with the association between 6PPD-Q formation and vehicle tire wear [63]. Inhalation of 6PPD-Q adsorbed onto PM_2.5_ is a major human exposure pathway, with an estimated daily intake ranging from 0.16 to 1.25 ng/(kg·d) [3]. Elevated concentrations in high-traffic areas may therefore raise health concerns for chronically exposed individuals who live or work nearby [3].

Therefore, to mitigate these environmental and health concerns, targeted strategies are needed—such as optimizing tire materials and compositional ratios, improving tire wear resistance to reduce abrasion, and promoting low-carbon and sustainable modes of transportation. These measures could effectively decrease 6PPD-Q levels in the atmosphere and road dust, thereby reducing human exposure to this emerging pollutant.

### 3.3. Light

Following its release into ecological systems, 6PPD can undergo multiple transformation pathways leading to the formation of 6PPD-Q. Photochemical reactions between nitrogen oxides and volatile organic compounds generate gaseous ozone under light conditions, particularly in the presence of ultraviolet radiation [67]. When 6PPD is exposed to gaseous ozone, it can be oxidized to 6PPD-Q through intermediate pathways involving hydroxyl and semiquinone radicals [40]. In addition, 6PPD could be converted into 6PPD-Q via the *N*-1,3-dimethylbutyl-*n*-phenylquinone diamine (QDI) pathway [12].

Light, therefore, represents a key environmental factor influencing both the concentration and conversion rate of 6PPD-Q. Sunlight and elevated temperatures facilitate the transformation of 6PPD into 6PPD-Q, with higher temperatures generally correlating with higher 6PPD-Q concentration. In a recent simulated sunlight experiment, 6PPD was converted to 6PPD-Q within 90 min at pH 7.0, with a molar yield of approximately 1.01% [44]. Environmental concentrations of 6PPD-Q also exhibit seasonal variations associated with light intensity and temperature. Enhanced solar radiation and elevated in-vehicle temperatures during summer accelerate ozone-mediated reactions, thereby promoting 6PPD-Q formation [4]. In contrast, both 6PPD and 6PPD-Q levels in road dust and indoor parking lots exhibited minimal seasonal variation between January and December, as reduced sunlight exposure and lower ambient temperatures during winter suppress the oxidation of 6PPD [28,33]. Similarly, road dust samples from Tokyo, Japan displayed higher 6PPD-Q levels in May and June (790–1284 ng/g) compared with other months (366–900 ng/g), likely due to elevated temperatures and ozone levels during that period [1]. According to Zhang et al. [24], indoor office environments contain lower 6PPD-Q concentrations than semi-enclosed or outdoor areas, mainly due to both reduced initial 6PPD levels and limited ozone exposure, which inhibit the oxidation of 6PPD.

However, while sunlight promotes the conversion of 6PPD-Q from 6PPD, it simultaneously accelerates the degradation of 6PPD-Q itself. Under natural light-aging conditions, the half-life of 6PPD-Q is less than one month, whereas under dark thermal-aging conditions, degradation slows significantly, resulting in a half-life of approximately six months [68].

These findings highlight that light simultaneously facilitates the transformation and degradation of 6PPD-Q, making its environmental behavior strongly dependent on the interplay between light intensity and temperature. Regional variations in these factors likely explain the consistently higher 6PPD-Q concentrations compared to 6PPD observed in Korean road dust collected from inland streams and areas influenced by industrial and traffic emissions [54]. Nevertheless, the specific mechanisms governing 6PPD-Q behavior under varying light conditions remain poorly understood, highlighting the need for future research to elucidate its transformation pathways, persistence, and fate across diverse illumination regimes.

### 3.4. Particle Size of Particulate Matter

The distribution of pollutants in dust varies significantly with particle size [69,70]: fine particles possess greater mobility and are more easily dispersed in the air compared to coarse fractions. Both Deng et al. [28] and Hiki et al. [1] found that the fine fraction contained higher concentrations of 6PPD and 6PPD-Q than the coarse fraction (>125 μm). This enrichment pattern is likely attributed to the larger specific surface area of fine particles, which facilitates the adsorption of organic pollutants onto particle surfaces and leads to elevated contamination levels [71]. These findings are consistent with observations from road tunnel dust samples, where finer particles (<100 μm) were found to contain higher concentrations of 6PPD and 6PPD-Q compared to coarser fractions [30].

It is important to note, however, that the particle-size-dependent distribution of 6PPD-Q in atmospheric particulate matter shows a different but complementary trend to that observed in settled dust. While fine dust particles tend to accumulate higher levels of 6PPD-Q due to surface-area effects, in the atmosphere, coarser airborne particles can act as the dominant carriers of particle-bound 6PPD-Q. This difference arises from the distinct physicochemical behaviors and aerodynamic properties governing airborne particulates [72]. The log K_OA_ of 6PPD-Q is 15.319 (above 10), suggesting low volatility and a strong tendency for adsorption and deposition onto airborne particles [27]. Size-segregated particulates have revealed that 6PPD-Q tends to accumulate on larger airborne particles within specific particle size ranges. Specifically, the concentration of 6PPD-Q ranged from 1.04 to 3.73 pg/m^3^ in particles of 1.1–2.1 μm, whereas it reached 7.78–23.2 pg/m^3^ in those of 9–10 μm [24]. Similarly, Jiang et al. [73] reported that 6PPD-Q preferentially associates with particles in the 4.7–9.0 μm range. Therefore, although finer fractions in dust exhibit higher contamination levels, coarser airborne particles may dominate the atmospheric transport and deposition of 6PPD-Q under certain conditions.

The deposition site and extent of particulate matter in the human respiratory system is a key determinant of its size distribution; concurrently, the toxicological outcomes—whether toxic, carcinogenic, or mutagenic—are critical in determining its chemical composition [74]. Therefore, to reliably assess the health risks of particulate-bound 6PPD-Q exposure, further investigation into the size-dependent respiratory deposition behavior of 6PPD-Q is urgently needed.

## 4. Toxic Effects

### 4.1. Bioaccumulation

6PPD-Q can bioaccumulate in organisms and undergo trophic transfer through the food chain. With an octanol-water partition coefficient (log K_ow_) of 3.41 (data from ChemSpider) under natural conditions, it exhibits a strong potential for accumulation in living organisms. For instance, after 14 days of exposure to 1 mg/L solutions of 6PPD and 6PPD-Q, cumulative concentrations in hydroponic lettuce reached 0.75 μg/g and 2.19 μg/g, respectively [75]. Ji et al. [76] were the first to document the presence of 6PPD and 6PPD-Q in fish species, revealing that 6PPD-Q bioaccumulates in the gills and brain of marine species [77] and may further biomagnify through food chains.

Experimental studies in laboratory mice have also demonstrated systemic distribution and toxicity of 6PPD-Q. The compound was detected in multiple organs—including the kidneys, lungs, testes, liver, spleen, heart, and skeletal muscle—and was shown to compromise the integrity of the blood–brain barrier [78]. A single oral dose of 4 mg/kg 6PPD-Q caused marked histopathological injury in the liver, spleen, lungs, and cerebral cortex, and renal cortex. Exposure to a single 4 mg/kg dose of 6PPD-Q was sufficient to elicit discernible histopathological alterations in the mouse liver, spleen, lungs, cerebral cortex and renal cortex. Repeated administration of the same dose aggravated these pathological changes, indicating cumulative organ injury and bioaccumulation potential [79]. Moreover, significant accumulation of 6PPD-Q was observed in adipose tissue, with concentrations peaking at 12 h (262 ± 31.4 ng/g) and remaining elevated after 72 h (18.0 ± 1.4 ng/g) [78]. This pattern reflects its lipophilic nature, consistent with its high partitioning into lipid-rich tissues, where fat acts as a long-term reservoir for lipophilic contaminants [79].

Furthermore, 6PPD-Q exhibits a greater bioaccumulation potential than 6PPD, likely due to its slower in vivo degradation. For instance, urinary excretion levels of 6PPD were higher than those of 6PPD-Q in mouse studies, suggesting reduced elimination and enhanced persistence of 6PPD-Q in organisms [80].

### 4.2. Aquatic Life

#### 4.2.1. Fish

6PPD-Q is widely detected in runoff samples, where it infiltrates water bodies and poses a threat to aquatic ecosystems [10,21]. It has been identified as a principal factor contributing to urban runoff mortality syndrome in coho salmon [10]. To date, research on the aquatic toxicity of 6PPD-Q has focused primarily on fish, where pronounced interspecific variability in sensitivity has been observed. Comparative toxicity studies revealed striking species differences: juvenile coho salmon were highly susceptible (12 h LC_50_ = 80.4 ng/L), whereas sockeye salmon showed no mortality, and Chinook salmon exhibited effects only at concentrations exceeding 25 μg/L [81]. Further investigation comparing newly feeding juvenile chinook and coho under static 24 h exposure confirmed this disparity, with LC_50_ values of 41.0 ng/L for coho and over 67 μg/L for Chinook—indicating nearly a three-order magnitude difference in sensitivity [82]. Both species showed typical symptomology, including gasping, rapid ventilation, erratic swimming, and loss of equilibrium, with symptomatic fish generally succumbing to mortality [82]. Consistent with these findings, Brinkmann et al. [83] reported significant mortality in brook trout and rainbow trout at 0.59 μg/L (24 h exposure) and 1.00 μg/L (72 h exposure), respectively, accompanied by abnormal behavior and elevated blood glucose levels. These effects were accompanied by clinical signs including increased ventilation rate. In contrast, white sturgeon, Atlantic salmon, and brown trout exhibited no mortality or behavioral effects even at concentrations up to 14.2 μg/L [84]. Moreover, exposure to environmentally relevant concentrations (0.1–10 μg/L) triggered lethal and developmental impairments in coho salmon, compromising their survival, growth, and morphological development [85].

Toxicity studies have also demonstrated pronounced life-stage variability in sensitivity to 6PPD-Q. Early life stages of species, such as alevins, embryos, and fry, are more vulnerable to 6PPD-Q at lower concentrations. For example, lake trout and rainbow trout alevins exposed to environmentally relevant concentrations of 6PPD-Q experienced high mortality, with 45-day LC_50_ values at 0.39 μg/L, accompanied by developmental deformities such as blood pooling in fins and eyes. Similarly, zebrafish embryos exposed to 25 μg/L 6PPD-Q during early development (0–96 h post-fertilization) exhibited marked morphological abnormalities, behavioral toxicity, and cardiotoxicity in zebrafish [82]. These adverse effects are characterized by reduced spontaneous movement, increased malformation rate, and decreased heart rate [86]. Age-related differences have also been observed in salmonids. The LC_50_ for juvenile coho (41.0 ng/L) [82] was approximately 2.3-fold lower than that reported for older (>1 y) individuals (95 ng/L) [87], highlighting the heightened vulnerability of younger life stages. Additional studies determined 24 h and 96 h LC_50_ values of 308.67 μg/L and 132.92 μg/L, respectively [88]. In contrast, Varshney et al. [89] observed no mortality in adult zebrafish following a 96 h exposure to 50 μg/L 6PPD-Q, suggesting that mature individuals exhibit substantially greater tolerance to this compound.

Table 3 presents the acute toxic effects of 6PPD-Q across various species. While Considerable research has focused on the acute toxicity of 6PPD-Q, the chronic effects remain largely unexplored and are not yet fully understood. Consequently, future studies should prioritize examining species-specific toxicity responses in aquatic ecosystems and systematically evaluate the long-term risks posed by 6PPD-Q contamination.

**Table 3:** Summary of the acute toxicity of 6PPD-Q to various aquatic species.

Species	Duration of Exposure	LC_50_	References
Coho Salmon (>1 y)	24 h	95 ng/L	[87]
Coho Salmon (~3 weeks post swim-up)	24 h	41.0 ng/L	[82]
Chinook Salmon (~3 weeks post swim-up)	24 h	>67.307 μg/L	[82]
Coho Salmon (189 d)	12 h	80.4 ng/L	[81]
Lake Trout (8 weeks post hatch)	96 h	0.50 μg/L	[90]
Rainbow Trout (~2 y)	72 h	1.00 μg/L	[82]
Rainbow Trout (fry)	96 h	0.47 μg/L	[91]
Brook Trout (~1 y)	24 h	0.59 μg/L	[83]
Zebrafish (larval)	24 h	308.67 μg/L	[88]
	96 h	132.92 μg/L	[88]

#### 4.2.2. Invertebrates

Current knowledge of 6PPD-Q toxicity in aquatic invertebrates remains limited. Chronic 21-day exposure studies in daphnids have consistently demonstrated that 6PPD-Q induces sublethal ecological effects, even at low concentrations. For example, *Daphnia pulex* exposed to 0.1–10 μg/L exhibited growth inhibition without increased mortality [92], whereas *Daphnia magna* exposed to up to 20 μg/L displayed broader physiological impairments, including disruption of ecdysteroid and juvenile hormone pathways, growth retardation, reproductive inhibition, and activation of antioxidant defenses [93]. These findings collectively suggest that 6PPD-Q poses a sublethal risk by perturbing multiple physiological processes, potentially forcing a resource trade-off in energy allocation under stress. Marked interspecific differences have also been reported: the copepod *Acartia tonsa* showed no adverse effects even at 1000 μg/L, while echinoderm embryos (*Arbacia lixula* and *Paracentrotus lividus*) were highly sensitive, with EC_50_ values of only 7–8 μg/L [94]. Acute toxicity tests further revealed no lethal effects on *Daphnia magna* or *Hyalella azteca* at near-maximum solubility (~100 μg/L), in sharp contrast to coho salmon, which are approximately 100-fold more sensitive [95]. Collectively, these findings underscore the pronounced species-specificity of 6PPD-Q toxicity and highlight the need for broader evaluations across invertebrate taxa.

#### 4.2.3. Microalgae

6PPD and 6PPD-Q also impact microalgae, though their effects differ by species and concentration. For *Selenastrum capricornutum*, low levels of 6PPD (1–5 mg/L) stimulated growth, whereas higher concentrations (10–50 mg/L) inhibited growth, with a 96 h IC_50_ of 8.78 mg/L, and caused cellular damage such as increased membrane permeability and cell wall rupture. In contrast, 6PPD-Q showed no toxicity to this species at concentrations up to 10 mg/L [96]. Conversely, in *Chlorella vulgaris*, exposure to 6PPD and 6PPD-Q (50–400 μg/L) revealed that 6PPD-Q enhanced photosynthetic efficiency and promoted growth at lower concentrations (50–200 μg/L), but inhibited growth at 400 μg/L [97]. Moreover, 6PPD-Q induced stronger oxidative stress than 6PPD, disrupting cell membrane integrity and mitochondrial function, thereby exhibiting higher overall toxicity. Collectively, these results highlight species-specific and concentration-dependent responses, underscoring the ecological risks of tire wear particle-derived chemicals to aquatic plants.

### 4.3. Terrestrial Life

Studies on the toxicity of 6PPD-Q to terrestrial organisms remain limited, yet emerging evidence underscores its multifaceted risks to soil ecosystems. In earthworms (*Eisenia fetida*), 28-day exposure to 10–5000 μg/kg dw soil led to bioaccumulation and oxidative damage. Multi-omics analyses revealed inflammation, immune dysfunction, metabolic disruption, and genetic toxicity [98]. At concentrations ≥ 1000 μg/kg, 6PPD-Q significantly suppressed intestinal microbial functions related to metabolism and information processing (*p* < 0.05), which were accompanied by weight loss and reduced survival, indicating that environmentally relevant concentrations pose substantial hazards to soil biota [98].

The toxic effects of 6PPD-Q on *Caenorhabditis elegans* are also pronounced. Chronic exposure to 6PPD-Q at environmentally relevant concentrations (0.1–100 μg/L) induces multisystem toxicity, affecting metabolic, neural, and reproductive functions. Exposure to 1–10 μg/L promotes lipid accumulation, characterized by elevated triglyceride levels, enlarged lipid droplets, and enhanced fatty acid synthesis, while suppressing β-oxidation and increasing monounsaturated fatty acyl-CoAs [99]. Neurotoxicity is evident, with exposure impairing locomotion and sensory perception, particularly at 1–10 μg/L. At 10 μg/L, 6PPD-Q induced severe neurodegeneration of dopaminergic, glutamatergic, serotonergic, and GABAergic neurons, leading to reduced neurotransmitter levels (dopamine, serotonin, glutamate, GABA) and disrupted neurotransmission-related gene expression. Molecular docking studies further suggest that 6PPD-Q directly binds to neurotransmitter synthesis proteins [100]. Dopamine metabolism disruption was also evident, with decreased dopamine levels, altered dopamine-related behaviors, and downregulation of dopamine synthesis genes (*cat-2*, *bas-1*) and the dopamine transporter gene (*cat-1*) [101]. Additionally, neurodegeneration of D-type motor neurons was observed in nematodes exposed to 10 μg/L of 6PPD-Q, linked to activation of the DEG-3-mediated Ca^2+^ channel signaling cascade [102]. In terms of reproductive toxicity, 6PPD-Q exposure (1–10 μg/L) reduced reproductive capacity and increased germline apoptosis, which was associated with upregulation of apoptosis-related genes (*ced-3*, *ced-4*, *egl-1*) and downregulation of the anti-apoptotic gene (*ced-9*). DNA damage and enhanced cell corpse engulfment further drive this toxicity [103]. Additionally, 6PPD-Q exposure (1–100 μg/L) triggers ferroptosis, characterized by elevated Fe^2+^, increased malondialdehyde (MDA), reduced glutathione (GSH), and enhanced lipid peroxidation, which is critically associated with reproductive toxicity [104]. Finally, parental exposure to 0.1–10 μg/L 6PPD-Q induced transgenerational lipid accumulation, driven by the persistent upregulation of fatty acid and monounsaturated fatty acyl-CoA synthesis genes and suppression of β-oxidation genes [105].

Further evidence of the adverse impact on soil invertebrates is provided by studies on *Folsomia candida*, where adult populations were negatively correlated with 6PPD-Q concentrations after 28 days of soil exposure [106]. In addition to fauna, 6PPD and 6PPD-Q can also be absorbed and metabolized by plants, with a maximum concentration of 0.78 μg/g detected in lettuce leaves, where 6PPD-Q was found to be more stable than 6PPD [75].

These findings highlight the urgent need to consolidate current knowledge on 6PPD-Q’s terrestrial ecotoxicology to better understand its environmental fate and impacts.

### 4.4. Human Exposure

The primary pathways for human exposure to 6PPD-Q include dietary intake, inhalation of dust, drinking contaminated water, and skin contact [3,22,67]. Cao et al. [23] found that ingestion of roadside soil dust was the primary source of human exposure to PPDs and PPD-Qs, with dermal absorption being the second-largest exposure pathway, contributing nearly 15% of intake from oral ingestion. Similarly, Geng et al. [11] assessed exposure to PPDs and PPD-Qs via water, environmental dust, and inhalation. Their findings showed that drinking water was the dominant exposure route, contributing 76.6–90.6% (mean 82.5%) of the total dose, while dust exposure accounted for 5.8% and 19.1% in adults and children, respectively. Dermal absorption of dust contributed 1.6–1.8% of total dust exposure in both groups. Cao et al. [23] also estimated the daily intake of PPDs and PPD-Qs for both adults and children in Hong Kong. For children, the total daily intake of PPD-Qs was estimated at 7.30 ng/(kg·d), slightly higher than that of PPDs [4.85 ng/(kg·d)]. In contrast, the daily intake for adults was lower, at 1.08 ng/(kg·d) for PPD-Qs and 0.71 ng/(kg·d) for PPDs. Zhu et al. [107] assessed the daily intake of 6PPD-Q via indoor dust exposure across three population groups (infants, children, and adults) in a case study conducted in Hangzhou, China. Infants exhibited the highest intake [0.85–259 (pg/kg bw/d)], followed by children [0.37–112 (pg/kg bw)/d] and adults [0.18–56 (pg/kg bw)/d], with infants experiencing relatively higher exposure due to their lower body weight. Additionally, physiological differences between children, particularly young children, and adults—such as variations in protein binding capacity, blood flow, and enzyme activity—can influence the absorption, distribution, metabolism, and excretion of 6PPD-Q [108,109]. Furthermore, Zhang et al. [110] compared 6PPD-Q intake in kindergarten children with varying body mass indexes (BMIs) and found that higher dust-borne 6PPD-Q intake correlated lower BMIs and increased incidence of influenza and diarrhea.

The presence of PPDs and PPD-Qs has been confirmed in human urine, serum, and cerebrospinal fluid, indicating uptake via the above-mentioned pathways and raising health concerns, such as lipid peroxidation damage, hepatotoxicity [111], and neuronal mitochondrial dysfunction [77]. For example, an analysis of 109 paired whole blood specimens from a general adult population in China detected 6PPD (<LOD–0.60 μg/L) and 6PPD-Q (<LOD–0.74 μg/L) in 39% and 43% of samples, respectively [112]. Pregnant individuals exhibited a median urinary 6PPD-Q concentration of 2.91 μg/L, significantly higher than in adults (0.40 μg/L) and children (0.076 μg/L), with mean urinary excretion reaching 273 (ng/kg bw)/d [113]. This may be due to a faster metabolic rate, resulting in higher renal clearance and urinary excretion of 6PPD-Q, as well as increased water consumption during pregnancy, which may further elevate exposure to the chemical [113]. Similarly, significant sex-based disparities (*p* < 0.05) were observed, with females excreting 7.381 (ng/kg bw)/d of 6PPD-Q, nearly double the 3.360 (ng/kg bw)/d excreted by males, suggesting that women may face higher daily exposure risks [114]. This disparity may be attributed to more frequent contact with rubber products and physiological differences between males and females. Further analyses showed that urinary 6PPD-Q excretion was correlated with biomarkers such as alanine aminotransferase, thyroid-stimulating hormone, and hematological parameters, revealing potential associations with liver function and immune responses [114]. In addition, elevated 6PPD-Q levels in the cerebrospinal fluid of Parkinson’s disease patients (11.18 μg/L) were more than double those found in healthy controls (5.07 μg/L), and untargeted metabolomics indicated a significant correlation between 6PPD-Q exposure and disturbances in neuronal metabolic profiles, suggesting its potential neurotoxicity [77].

To better understand how 6PPD-Q affects humans and its underlying mechanisms, mammals, particularly mice, are frequently employed as test organisms due to their physiological and biochemical similarities to humans. Research has shown that 6PPD-Q is quickly absorbed following oral administration, distributed through the blood to various organs, including the liver, brain, and placenta in pregnant mice after a five-day oral exposure period [80]. Toxicological studies have identified the liver represents the primary target organ in mice exposed to 6PPD-Q (70 ± 90 μg/kg), with significant increases in both liver weight and hepatic triglycerides, indicating hepatotoxicity [115]. As the main detoxification organ, the liver metabolizes and transforms 6PPD-Q, triggering various physiological responses, including hepatomegaly, lipid accumulation, hepatocellular damage, and apoptosis [116]. In vitro cytotoxicity assessments also indicated that PPDs (including DPPD, DNPD, and 6PPD) and PPD-Qs significantly reduced the survival of human hepatoma cells [117].

In addition to the direct health risks posed by 6PPD-Q, co-exposure to other environmental contaminants, such as heavy metals, particulate matter, and microplastics, can amplify its toxic effects through combined toxicity [26]. The persistence, mobility, and toxicity of 6PPD-Q in the environment highlight the need for continuous monitoring. Future studies should focus on assessing the chronic toxicity of prolonged, low-concentration exposure to 6PPD-Q, as well as its interaction with other contaminants. Further studies are essential to elucidate its toxicity mechanisms, improve exposure and risk assessments, and inform the development of effective mitigation strategies to protect human health and ecosystems. Additionally, it is crucial to establish toxicological threshold limits for human exposure to 6PPD-Q and set corresponding environmental standards.

## 5. Risk Control

To systematically prevent and control 6PPD-Q contamination, a comprehensive strategy encompassing “source reduction—process control—end-of-pipe treatment” must be adopted, thereby establishing a closed-loop management framework. At the source level, improving tire additive formulations may be an effective approach to mitigate the environmental hazards of TWPs [18]. Efforts should focus on developing and applying environmentally friendly antiozonants through the selection of raw materials with low impurity content and the optimization of rubber formulations to minimize 6PPD usage. However, eliminating 6PPD poses safety concerns, as tires without its protective function may fail after only 161–1610 km of driving [107]. Developing substitutes for 6PPD remains a major challenge for the industry, as rubber materials are highly vulnerable to degradation induced by reactive species such as peroxyl radicals, alkyl radicals, and ozone [118,119]. Therefore, current research priorities include the identification of substitute compounds for 6PPD or the design of novel rubber materials with lower degradation potential while maintaining tire performance and safety. Achieving this transition would not only effectively reduce the environmental release of 6PPD-Q from TWPs, but also foster greater sustainability within the tire industry.

The process control phase is critical for the timely management of generated TWPs to prevent and control their migration across environmental compartments [18]. Phytoremediation is an effective nature-based tool in this regard, as plants absorb gaseous pollutants through leaf stomata, thereby lowering ambient concentrations of harmful particulates [120,121,122]. Strategically planned vegetation buffers can therefore contribute to reducing TWP pollution [123]. For example, roadside vegetation with high deposition rates has been shown to significantly mitigate TWP emissions from passenger vehicles [124]. Given the bioaccumulation potential of 6PPD-Q, employing phytoremediation to reduce its environmental concentration can help mitigate its accumulation in top predators of the food chain. Future efforts should focus on identifying plant species with high uptake efficiency, quantifying their removal capacity—particularly for 6PPD-Q—and integrating phytoremediation into existing management strategies as a complementary solution.

Road sweeping is another practical approach, removing deposited TWPs with other road dust [125,126]. Particle size is a determining factor: lighter and smaller particles are more readily transported into stormwater sediments, whereas larger and heavier particles tend to remain on the road surface and can be collected via sweeping [127]. Sweeping efficiency is strongly influenced by vehicle operation, with studies indicating that lower sweeper speeds (4.7–10 km/h) achieve greater dust removal efficiency compared to higher speeds [128].

The end-of-pipe treatment phase primarily refers to WWTPs and stormwater management systems. Wastewater generated from road cleaning, which often contains high concentrations of TWPs, is initially transported through municipal pipelines, making the stormwater treatment systems a feasible measure for end-of-pipe management [18]. Studies have shown that bioretention basins and sedimentation processes can effectively intercept TWPs, with removal rates ranging from 55% to 100% [129]. For typical rainfall events with recurrence period of less than two years, stormwater bioretention basins have demonstrated >90% removal of 6PPD-Q [130]. Another promising strategy is upgrading existing WWTPs to enhance their capacity for TWP removal [18]. Treatment efficiency can be further improved through treatment process optimization, such as improving sedimentation efficiency and upgrading biological treatment units, thereby establishing a more robust multi-barrier defense system.

Overall, these findings highlight the importance of adopting a multi-tiered approach to address 6PPD-Q contamination. Source reduction, process control, and end-of-pipe treatment should be integrated within a broader framework that also considers ecological restoration and sustainable tire innovation. Future research should emphasize the identification of safer substitutes for 6PPD, the optimization of phytoremediation techniques, and the enhancement of stormwater and wastewater treatment systems. Such efforts will not only mitigate the environmental and health risks posed by 6PPD-Q, but also provide long-term strategies to promote sustainability in the automotive and tire industries.

## 6. Summary and Outlook

6PPD-Q, the oxidative transformation product of the tire antioxidant 6PPD, has emerged as a research hotspot in environmental science due to its widespread occurrence, high toxicity to organisms, and potential risks to ecosystems and human health. It is widely distributed in the atmosphere, water bodies, soils, and sediments, with concentrations influenced by precipitation patterns, traffic characteristics, light exposure, and particle size. 6PPD-Q demonstrates strong acute toxicity to organisms, with marked species-specific variations. Human exposure primarily occurs through diet, inhalation, and dermal contact, and has been linked to hepatotoxicity, neurotoxicity, and other adverse health effects.

To address these risks, a comprehensive “source reduction—process control—end-of-pipe treatment” framework has been proposed. At the source level, efforts focus on developing environmentally friendly antioxidants or novel tire materials to reduce 6PPD usage and subsequent 6PPD-Q formation. Process-level measures, including optimized road sweeping and phytoremediation, can help intercept tire wear particles (TWPs) and mitigate their accumulation in surrounding environments. End-of-pipe strategies, such as bioretention basins, sedimentation processes, and upgraded wastewater treatment plants, have demonstrated substantial removal efficiency for 6PPD-Q under typical urban rainfall and stormwater management conditions. Although considerable progress has been achieved, significant knowledge gaps remain. Further studies should emphasize simultaneous monitoring of 6PPD-Q in both the particulate and aqueous phases to better quantify environmental loads and human exposure risks. Greater attention is also needed on the chronic effects of long-term, low-dose exposure, as well as the combined effects of 6PPD-Q with co-occurring pollutants, such as heavy metals.

Looking ahead, interdisciplinary research that integrates environmental chemistry, toxicology, and engineering will be critical for developing systematic solutions—from molecular-level mechanisms to regional-scale management. Moreover, establishing environmental quality standards and toxicological thresholds for 6PPD-Q, as well as strengthening international cooperation to develop a unified risk assessment framework, is urgently required. In other words, advancing both scientific research and policy development is essential. Building a comprehensive risk management framework that couples scientific insight with technological innovation and regulatory support will be of great significance for protecting ecosystem integrity and human health while also guiding the tire industry toward a more sustainable future.

## Data Availability

No new data were created or analyzed in this study. Data sharing is not applicable to this article.

## Abbreviations

The following abbreviations are used in this manuscript:

6PPD	*N*-(1,3-dimethylbutyl)-*N*′-phenyl-*p*-phenylenediamine
6PPD-Q	*N*-(1,3-dimethylbutyl)-*N*′-phenyl-*p*-benzoquinone
TWPs	Tire wear particles
PPDs	*p*-Phenylenediamines
PPD-Qs	PPD-quinones
IPPD	*N*-isopropyl-*N*′-phenyl-*p*-phenylenediamine
44PD	*N*,*N*′-di-sec-butyl-*p*-phenylenediamine
77PD	*N*-(1,4-dimethylpentyl)-*N*′-phenyl-*p*-phenylenediamine
CPPD	*N*-cyclohexyl-*N*′-phenyl-*p*-phenylenediamine
DPPD	*N*,*N*′-diphenyl-*p*-phenylenediamine
DNPD	*N*,*N*′-di(2-naphthyl)-*p*-phenylenediamine
WWTPs	Wastewater treatment plants
QDI	*N*-1,3-dimethylbutyl-*n*-phenylquinone diamine
